# MicroRNA-7 as a Potential Biomarker for Prognosis in Pancreatic Cancer

**DOI:** 10.1155/2020/2782101

**Published:** 2020-06-01

**Authors:** Zhi-qiang Ye, Chang-lin Zou, Han-bin Chen, Ming-jie Jiang, Zhu Mei, Dian-na Gu

**Affiliations:** ^1^Department of Thyroid and Breast Surgery, The First Affiliated Hospital of Wenzhou Medical University, Wenzhou, Zhejiang 325000, China; ^2^Department of Chemoradiotherapy, The First Affiliated Hospital of Wenzhou Medical University, Wenzhou, Zhejiang 325000, China; ^3^Institute of Translational Medicine, Shanghai General Hospital, Shanghai Jiao Tong University School of Medicine, Shanghai 201620, China; ^4^Shanghai Key Laboratory of Pancreatic Diseases, Shanghai General Hospital, Shanghai Jiao Tong University School of Medicine, Shanghai 201620, China; ^5^Department of Gastroenterology, Shanghai General Hospital, Shanghai Jiao Tong University School of Medicine, Shanghai 201620, China

## Abstract

MicroRNAs play critical roles in tumor progression. Our recent study has indicated that microRNA-7 (miR-7) impairs autophagy-derived pools of glucose to suppress the glycolysis in pancreatic cancer progression. However, the roles of miR-7 in clinical significance and chemoresistance of pancreatic cancer remain unexplored. The aim of this study was to assess the expression of miR-7 in patients with pancreatic cancer and to evaluate the possibility of its usage as a prognostic molecular biomarker. MicroRNA array-based quantification analysis of 372 miRNAs was compared in serum between pancreatic cancer and healthy individuals, gemcitabine-sensitive and gemcitabine-resistance patients. We identified miR-7 showed the potential predictive power for gemcitabine-sensitive patients with pancreatic cancer. Then, the results were validated in pancreatic tissue microarray and The Cancer Genome Atlas (TCGA) dataset, demonstrating that lower miR-7 expression was correlated with more advanced tumor stages and worse prognosis in pancreatic cancer. The Cox proportional-hazards model analysis identified miR-7 to be an independent variable for prediction of the survival. Furthermore, the mechanistic exploration suggested the clinical significance of miR-7 involved its interference effect on autophagy and glycolysis in pancreatic cancer using pancreatic cancer tissue microarrays and TCGA data. Therefore, the results of the present study provide evidences that low microRNA-7 expression may contribute to tumor progression and poor prognosis in pancreatic cancer.

## 1. Background

Pancreatic ductal adenocarcinoma (PDAC) is the most aggressive primary pancreatic neoplasm and has the poorest prognosis among the solid tumor cancers. Despite of the dramatic progress in the treatment and understanding the molecular mechanisms of carcinogenesis, only approximately ~5% of patients with PDAC will live 5 years after the initial diagnosis [1]. The poor survival rate is attributed to the fact that the majority of patients are diagnosed at advanced disease stages, when pancreatic surgical resection is not possible. In addition, gemcitabine has been proved to improve the median survival time and quality of life in advanced pancreatic cancer patients [2]. Over the past decade, gemcitabine has been widely used as a standard first-line therapy for pancreatic cancer. However, the prognosis of the disease remains dismal. Thus, it is necessary to explore the biomarkers for early diagnosis, and also for the prediction of therapy efficacy and prognosis, that could inform decision-making, facilitating personalized treatment, and an optimal clinical outcome.

MicroRNAs (miRNAs), which are small noncoding RNAs that induce degradation or translational repression of target gene mRNA, are stable in tissues and blood plasma. Consequently, miRNAs are ideal molecules to be utilized as biomarkers. Emerging evidences suggest miRNAs are potentially involved in carcinogenesis, proliferation, and apoptosis, therefore functioning as tumor suppressors or oncogenes [3]. A large number of miRNAs have been proven to be aberrantly expressed and are associated with pancreatic cancer cell proliferation, survival, invasion, and metastasis [4], including miR-142, miR-196b, Let-7, and miR-23b. Therefore, more extensive investigations are required to identify the correlations between miRNAs and the clinical characteristics of PDAC and to clarify the roles of these miRNAs in PDAC.

miR-7 has been characterized as a potential tumor suppressor in hepatocellular carcinoma [5], gastric cancer [6], colorectal cancer [7], breast cancer [8], etc. and regulated diverse fundamental biological processes of cancer cells by targeting a number of oncogenic signaling pathways. Recently, we observed that miR-7 could impair autophagy-derived pools of glucose to suppress pancreatic cancer progression. miR-7 not only repressed tumorigenesis and metastasis of PDAC xenografts but also reduces tumor growth in the PDAC patient-derived xenograft (PDX) model [9]. Others reported miR-7 could target a number of oncogenic signaling pathways in pancreatic cancers [10, 11]. Thus, we wonder whether miR-7 expression is correlated with its clinical relevance and used as a novel biomarker for diagnosis and prognosis in pancreatic cancer.

In the present study, we analyzed the expression level of miR-7 in the serum of pancreatic cancer and healthy individuals, gemcitabine-sensitive and gemcitabine-resistance patients, and validated these results in pancreatic tissue microarray and TCGA dataset. Further, we performed a systemic and comprehensive functional analysis for the diagnostic and predictive values of miR-7 in pancreatic cancer. We concluded that miR-7 was a promising prognosis biomarker for PDAC patients, and targeting miR-7 might be a potential therapeutic strategy for the treatment of PDAC patients.

## 2. Methods

### 2.1. Patient Population and Study Design

The present study was conducted in Shanghai General Hospital, Shanghai Jiao Tong University School of Medicine. All PDAC patients were diagnosed by biopsy and were histologically confirmed according to the American Joint Committee on Cancer (AJCC). A total of 8 patients with pancreatic cancer (4 males and 4 females), aged 51~76 years (median = 62.5 years) between 2011 and 2012, who received gemcitabine monotherapy without interventional therapy or radiotherapy, were included in this study and retrospectively examined for the predictive/prognostic significance of miR-7 expression. The clinical stage was evaluated using the International Union Against Cancer TNM classification. Patients were excluded if they had received radiotherapy or chemotherapy or had a previous history of other cancer. The efficacy was assessed every two courses of chemotherapy. Patients were followed-up for six courses of gemcitabine treatment. After completed evaluation, the study population consisted of 11 samples: one group of PDAC patients (*n* = 8) and one group of healthy controls (*n* = 3). For each participant, 5~6 ml of fasting blood was collected into BD Vacutioner® Citrate Tubes and centrifuged immediately. Then, the cell-free fractions were stored at -80°C. This study was approved by the Ethics Committee of Shanghai General Hospital, Shanghai Jiao Tong University School of Medicine. Participants signed a written informed consent before the study.

### 2.2. miRNA qRT-PCR Microarray Assays

miRNA expression profiling was performed by qRT-PCR. In brief, total RNA was isolated from plasma samples using the miRNeasy Serum/Plasma Advanced Kit (QIAGEN) according to the manufacturer's instructions. RNA was reverse transcribed and then assayed in 10 *μ*l PCR reactions using the miRCURY LNA™ Universal RT miRNA PCR, polyadenylation, and cDNA synthesis kit (Exiqon). miRNA was assayed by quantitative polymerase chain reactions (qPCR) using the miRCURY LNA™ Universal RT microRNA PCR, Ready-to-Use Human Panel I V4, which contains 372 specific miRNA primers. cel-miR-39-3p was used as RNA spike-in control to normalize miRNA expression levels. Amplification was performed in a QuantStudio 6 Flex Real-Time PCR System (Life Technology).

### 2.3. Tissue Microarrays

Pancreatic tissue microarrays (HPan-Ade060CS-01 and HPan-Ade180Sur-02) were purchased from Shanghai Outdo Biotech Co., Ltd. HPan-Ade060CS-01 included 29 cases of pancreatic ductal adenocarcinoma tissues and paired adjacent nontumor tissues and 2 of normal pancreatic tissues. HPan-Ade180Sur-02 incorporated 100 cases of pancreatic tumor and 80 of adjacent nontumor tissues. All the raw data are available from Shanghai Outdo Biotech Co., Ltd.

### 2.4. Measurement of Cell Viability and Colony Formation *In Vitro*

The human pancreatic ductal adenocarcinoma cell lines BxPC-3, MIA PaCa-2, and PANC-1 were obtained from the Cell Bank of Chinese Academy of Science (Shanghai, China) in June 2017. Cell lines were tested for mycoplasma contamination by the Mycoplasma qPCR Detection Kit (Genechem, Shanghai, China). Cells were not further tested or authenticated by the authors and passaged for <6 months after receipt or resuscitation for this study. In the previous study, it identified that pancreatic cancer cell lines BxPC-3 was sensitive, but MIA PaCa-2 and PANC-1 were resistant to gemcitabine *in vitro* [12]. BxPC-3, MIA PaCa-2, or PANC-1 cells were trypsinized into a single-cell suspension and seeded in 96-well or 6-well plates at 48 h post transiently transfected with miR-7 mimics or negative control. Then, pancreatic cancer cells were treated with gemcitabine at the concentration of 0.01, 0.1, 1, or 10 *μ*M for 24 h [13]. We added 10 *μ*l of the CCK8 solution (Dojindo, Kumamoto, Japan) to each well of the 96-well plate (100 *μ*l/well cell suspension) for a 2 h incubation period at 37°C, and the absorbance rates were read at 450 nm by a microplate reader (Epoch2, BioTek). For colony formation assay, 10 d of incubation, the colonies were stained with 0.1% crystal violet dye.

### 2.5. *In Situ* Hybridization and Immunohistochemistry

*In situ* hybridization was performed using a 5′- and 3′-digoxigenin- (DIG-) labeled locked nucleic acid- (LNA-) based probe specific for miR-7-5p (E38915; Exiqon, Denmark). The probe was detected using antidigoxigenin-AP (11093274910; Roche) and NBT/BCIP ready-to-use tables (11697471001; Roche). The hybridization was conducted according to the manual of the miRCURY LNA™ microRNA Array Kit (Exiqon, Denmark) using a hybridization station (ThermoBrite).

Immunohistochemistry was performed on tissue microarray chips. The slides were probed with the following primary antibodies: rabbit anti-human LDHA (ab47010; Abcam) and rabbit anti-human LC3 (12741; Cell Signaling Technology), and then incubated with HRP-conjugated goat anti-rabbit secondary antibody. The proteins were visualized *in situ* with DAB chromogenic substrate. The results of immunostaining and hybridization were evaluated according to the reference [14].

### 2.6. TCGA RNA-seq Analysis

Raw RNA-seq data for 178 pancreatic cancer tumors and 4 normal pancreatic tissues were downloaded from the TCGA pancreatic ductal adenocarcinoma project (http://cancergenome.nih.gov). Data was analyzed for differential gene expression using limma 3.26.9. Normalized counts, *P* values, adjusted *P* values, fold expression change, and average expression for each gene were exported.

### 2.7. Statistical Analysis

Normally distributed data were presented as mean ± SD. Differences between means were assessed using Student's *t* test. Frequencies of categorical variables were compared using the Chi-square test. Cumulative probabilities of overall survival were computed with the Kaplan-Meier method, and the log-rank test was used to assess their statistical significance. The relationship between miR-7 and LDHA expression and miR-7 and LC3 expression in pancreatic cancer tissue microarray was assessed using Spearman's rank correlation. Statistical analyses were performed using SPSS 19.0 software. All statistical tests were 2 tailed, and *P* < 0.05 was considered to be statistically significant.

## 3. Results

### 3.1. miR-7 Is Significantly Lower Expressed in Gemcitabine-Resistant Pancreatic Cancer Patients

**Figure 1:**
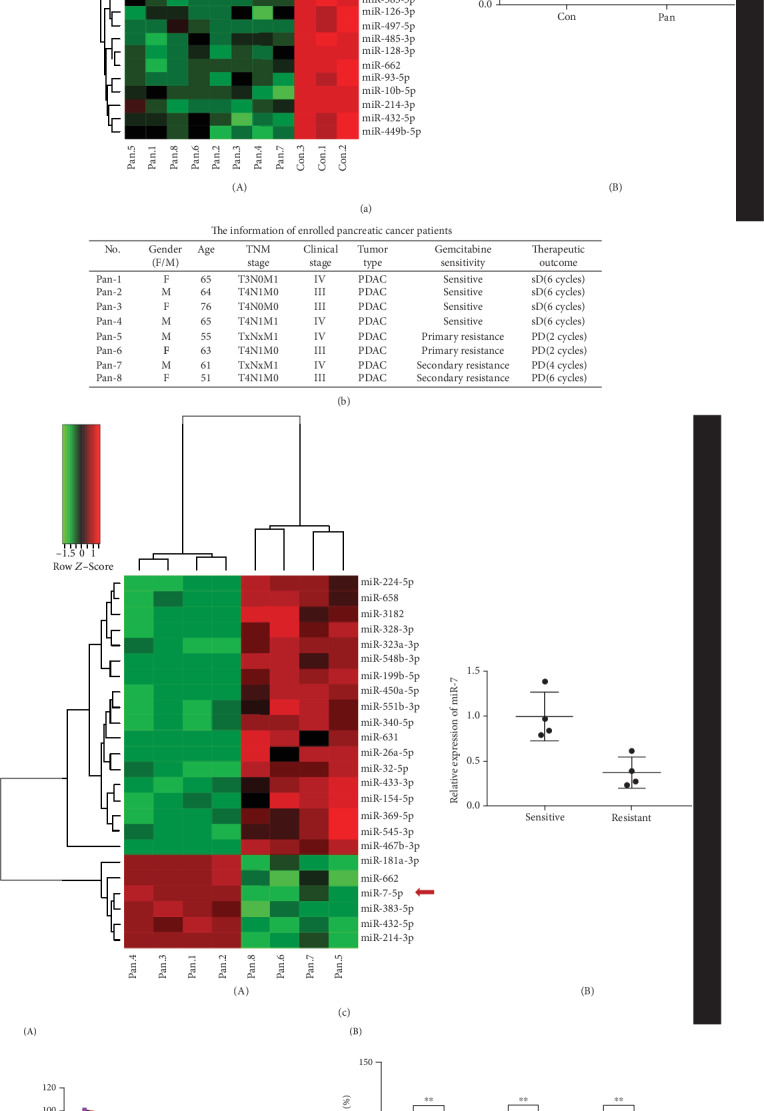
miRNA expression profiles in the plasma of pancreatic cancer and gemcitabine-resistance samples by statistical analysis of miRNA qRT-PCR array. (a) Heat map of miRNA expression in the serum of pancreatic cancer patients (*n* = 8) and healthy controls (*n* = 3) was presented in (A). The expression of miR-7 in pancreatic cancer patients (*n* = 8) and healthy controls (*n* = 3) was measured by qRT-PCR (B). (b) The information of enrolled pancreatic cancer patients. (c) Hierarchical clustering of miRNAs in the serum of the gemcitabine-sensitive group (*n* = 4) and the gemcitabine-resistance group (*n* = 4) was shown in (A). The miR-7 level of the gemcitabine-sensitive group (*n* = 4) was compared to that in the gemcitabine-resistance group (*n* = 4) (B). (d) Upregulation of miR-7 sensitized pancreatic cancer cells to gemcitabine. The pancreatic cancer cells were treated with gemcitabine for 24 h after transfected with miR-7 mimics. The viability of the cells was determined by CCK8 assay (A). BxPC-3, MIA PaCa-2, and PANC-1 were treated with gemcitabine (0.1 *μ*M for BxPC-3, 1 *μ*M for MIA PaCa-2, and PANC-1) for 24 h. Assessment of colony formation was performed when upregulation of miR-7 expression (B). (^∗∗^*P* < 0.01).

To identify circulating miRNA expression profiles in pancreatic cancer, expression levels of 372 miRNAs were measured by qRT-PCR technology in 11 serum samples (8 pancreatic cancer patients and 3 healthy controls). Among these 372 miRNAs, Student's *t* test identified 28 miRNAs that were significantly differently expressed in PDAC compared to healthy controls with a *P* value < 0.05 (more than 2-fold): 25 miRNAs were downregulated, while 3 miRNAs were upregulated (Figure 1(a), A). Furthermore, to the end point of follow-up, stable disease (SD) was observed in 4 and progressive disease (PD) was also observed in 4, which were enrolled in the gemcitabine-sensitive group or the gemcitabine-resistance group, respectively. Clinicopathological characteristics of recruited participants are listed in Figure 1(b). Compared with the gemcitabine-sensitive group, 24 miRNAs showed statistically significant changes (more than 2-fold) in the gemcitabine-resistance group: 6 of them were downregulated and the other 18 miRNAs were overexpressed (Figure 1(c), A). Hereinafter, we focused on miR-7. The expression level of miR-7 was not only decreased in pancreatic cancer compared to healthy controls (Figure 1(a), B) but also downregulated in the gemcitabine-resistance group compared to the gemcitabine-sensitive group (Figure 1(c), B).

To validate the effect of miR-7 on cell sensitive to chemotherapy, we treated pancreatic cancer cells with various concentrations of gemcitabine in the presence of either negative control or miR-7 mimics. After treating with gemcitabine, CCK8 assay showed that the viability was lower in miR-7 mimic-transfected cells than that in controls (Figure 1(d), A). Also, colony formation assay indicated that overexpression of miR-7 significantly reduced the number and size of colonies inoculated after 10 d compared with the control group (Figure 1(d), B). Thus, miR-7 could enhance the sensitivity of pancreatic cancer to gemcitabine.

### 3.2. Low miR-7 Correlates with Advanced PDAC Stage and Poor Prognosis

**Figure 2:**
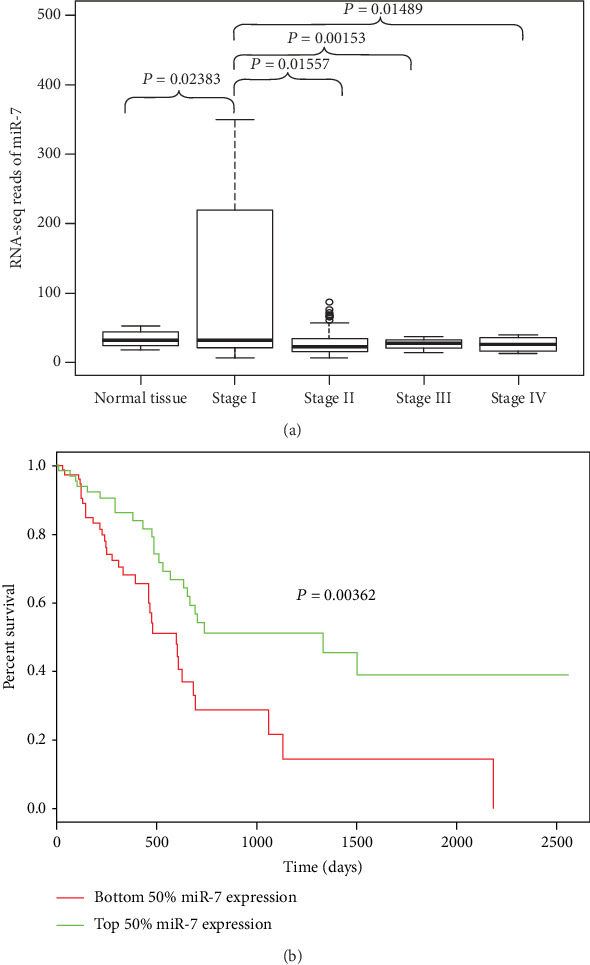
Lower miR-7 expression is correlated with the advanced tumor stage and worse patient prognosis from the TCGA database. (a) RNA-seq reads of miR-7 were normalized and categorized in 4 normal pancreas tissues and 178 pancreatic cancers from TCGA database. The expression level of miR-7 was examined in cancer tissue from pancreatic cancer patients with I~IV stage. (b) Kaplan-Meier analysis was performed to classify the 178 pancreatic cancer patients enrolled in TCGA database according to the expression of miR-7 in tumors (top and bottom 50% miR-7 expression).

We further explored the possible relationships between miR-7 and pancreatic cancer in clinical samples. At first, we assessed the diagnostic and prognostic values of miR-7 in pancreatic cancer from The Cancer Genome Atlas (TCGA) dataset. It demonstrated that lower miR-7 expression was significantly correlated with the advanced tumor stage (Figure 2(a)) and the worse patient prognosis (Figure 2(b)).

**Figure 3:**
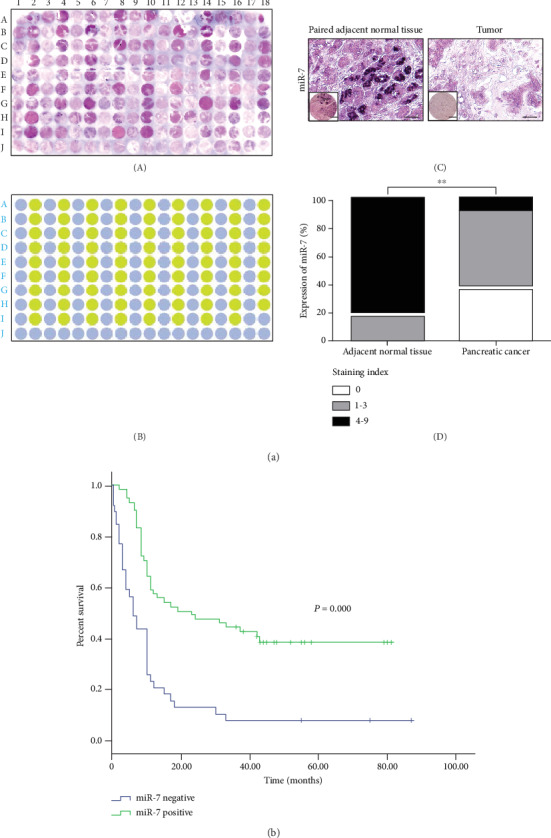
miR-7 is decreased in pancreatic cancer tissue and correlates with poor prognosis. (a) Pancreatic tissue microarrays were used for profiling miR-7 expression by *in situ* hybridization (A). The pareto diagram of the array was correspondingly shown (B) (blue dot, tumor; green dot, normal tissue). The expression of miR-7 in PDAC and adjacent normal tissues were representatively shown ((C) scale bar: 50 *μ*m (main); 250 *μ*m (inset)) and assessed semiquantitatively by staining intensity ((D) ^∗∗^*P* < 0.01). (b) Kaplan-Meier curves were used to show the overall survival of PDAC patients according to the expression of miR-7.

To validate the above result, we examined the expression of miR-7 by *in situ* hybridization in tissue microarrays containing 100 cases of pancreatic tumor and 80 of adjacent nontumor tissues (Figure 3(a), A, B). We observed that repression of miR-7 (staining index = 0) was detected in approximately 37% of the examined PDAC tumors (*n* = 100) compared to none of the adjacent nontumor tissues (*n* = 80) (Figure 3(a), C, D).

In addition, 100 cases of PDAC tissues were divided into two groups: a positive miR-7 expression group and a negative miR-7 expression group. Kaplan-Meier analysis indicated that the loss of miR-7 was associated with the poor prognosis of pancreatic cancer (Figure 3(b)). Furthermore, the analysis of clinical pathological characteristics showed that low miR-7 expression was significantly associated with poor tumor differentiation (*P* = 0.015), advanced TNM stage (*P* = 0.028), and distant metastasis (*P* = 0.011), but not with patient age, gender, tumor size, and lymphovascular invasion in pancreatic cancer (Table 1).

Notably, Cox proportional hazards regression modeling analysis showed that miR-7 expression independently predicted better survival in pancreatic cancer (HR = 0.325, *P* < 0.01) (Table 2). These results demonstrated that lower miR-7 expression levels indicated poorer prognosis in patients with pancreatic cancer.

### 3.3. miR-7 Repressed Autophagy and Glycolysis in Pancreatic Cancer

**Figure 4:**
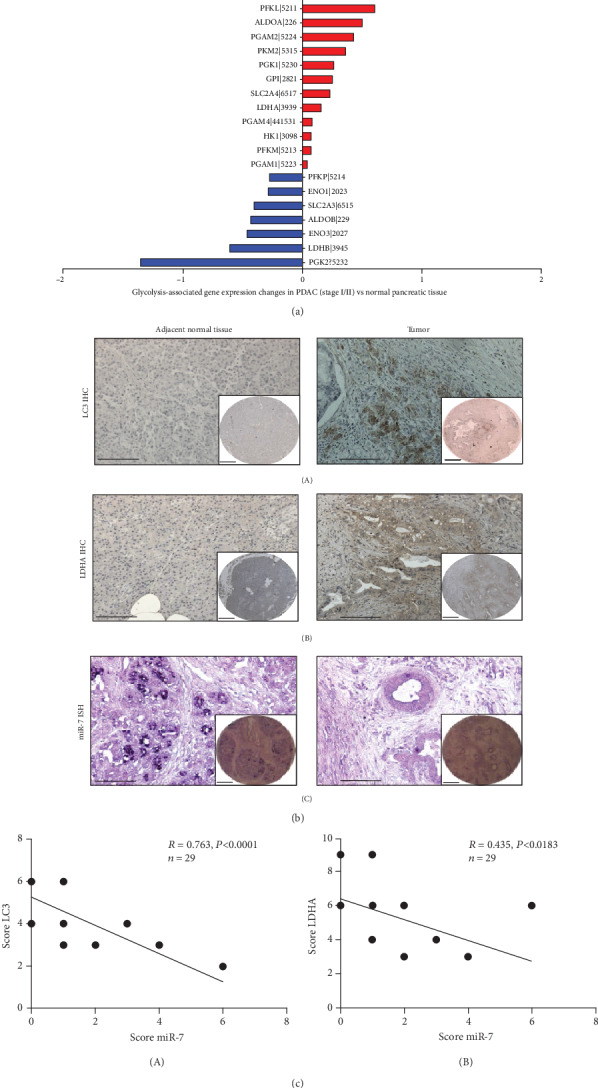
miR-7 inhibits autophagy and aerobic glycolysis in pancreatic cancer. (a) RNA-seq data from TCGA pancreatic ductal adenocarcinoma project was applied to analyze the expression of glycolysis genes. (b) Representative images of immunohistochemistry staining for LC3 and LDHA and *in situ* hybridization for miR-7 in pancreatic tissue microarray were shown. Scale bar: 100 *μ*m (main); 250 *μ*m (inset). (c) Relationship between miR-7 and LC3 expression (A) and miR-7 and LDHA (B) in pancreatic tissue microarray.

Our previous study has demonstrated that miR-7 could inhibit PDAC progression by repressing the activity of aerobic glycolysis via targeting autophagy *in vitro* and *in vivo* [9]; herein, we further explored the possible role of miR-7 in clinical pancreatic cancer samples. After analysis of the metabolic gene sets in PDAC RNA-seq data of TCGA, the glycolytic metabolism group was revealed significantly overexpressed (Figure 4(a)).

Further, in pancreatic cancer tissue microarrays, LC3 was detected to accumulate in the examined PDAC tumors (*n* = 29, Figure 4(b), A) compared to the adjacent normal tissues. Similar results were observed for glycolytic enzyme LDHA (*n* = 29, Figure 4(b), B). Nevertheless, miR-7 in pancreatic cancer samples was shown downregulated compared with their adjacent normal tissues (*n* = 29, Figure 4(b), C). Spearman correlation analysis revealed a negative correlation not only between miR-7 and LC3 but also between miR-7 and LDHA (Figure 4(c)). Collectively, in clinical pancreatic cancer patients, we also could observe miR-7 played an important role in preventing autophagy from supporting glycolysis in PDAC progression.

## 4. Discussion

Pancreatic cancer conveys unique tumor biology and complex microenvironment with hypoxia and nutrition deficiency which are distinct from most of other malignancies. Particularly, pancreatic cancers manifest high activity of autophagy and Warburg effect (also known as aerobic glycolysis). Recently, several studies have revealed that chemotherapeutic agent combinations targeting autophagy or glycolysis enhanced the treatment efficacy, which expands our view to explore a suitable biomarker based on the unique hallmarks of pancreatic cancer.

Circulating miRNAs stably exist in blood serum, providing a readily accessible and minimally invasive source for biomarker testing. In the current study, we first screened a large number of circulating microRNAs via real-time PCR microarrays, which enabled us to have a better chance to identify potential diagnostic markers. As a result, 28 miRNAs significantly differed in the plasma of healthy individuals from that of patients with pancreatic cancer. Meanwhile, since chemoresistance was one of the causes of poor prognosis in pancreatic cancer patients, we identified a panel of miRNAs dysregulated in gemcitabine-resistance pancreatic cancer. As a consequence, we found miR-7 to be decreased in serum from pancreatic cancer patients when compared with healthy controls, and also, lower miR-7 expression was associated with the resistance of pancreatic cancer to gemcitabine. Thus, we identified miR-7 might possess diagnostic and predictive potentials in pancreatic cancer.

miR-7 is primarily known to act as a robust tumor suppressor. The significance of miR-7 in cancer is well-documented to directly inhibit a number of oncogenic targets in multiple types of tumors, including hepatocellular carcinoma, gastric cancer, and colorectal cancer, and impede various aspects of cancer progression, including cell proliferation, invasion, and metastasis [15]. Successive studies conducted in colorectal cancer [16], thyroid cancer [17], prostate cancer [18], and breast cancer [8] identified miR-7 to be a useful biomarker for malignance, predicting prognosis or metastasis on the basis of blood serum or tissues. Moreover, recent emerging evidence indicates that circular RNA (ciRS-7), as a potential miR-7 sponge, could be a promising prognostic biomarker and a potential therapeutic target in colorectal cancer [19].

Heretofore, our results for the first time demonstrated that miR-7 showed great potentials as diagnostic and prognostic biomarkers for pancreatic cancer in clinical individuals. Evidences are presented as follows: Firstly, using tissue microarray, we further found that miR-7 decreased in most pancreatic tumors compared to normal pancreatic tissues. Moreover, a lower miR-7 level in pancreatic cancer patients was associated with a lower survival rate compared to higher miR-7. Besides, analysis of clinical data indicated that reduced expression of miR-7 in pancreatic cancer patients correlated with tumor differentiation, advanced TNM stage, and distant metastasis. Furthermore, Cox's multivariate analysis indicated that miR-7 expression, TNM stage, and distant metastasis acted as an independent factor in the prediction of overall survival among patients with PDAC, respectively. Consistently, TCGA pancreatic cancer RNA-seq data analysis indicated that lower miR-7 expression was strongly correlated with more advanced tumor stages and worse prognosis. Taken together, these data proved miR-7 could be a potential biomarker for pancreatic cancer.

Mechanistically, to better appreciate the biological significance of miR-7 for its contribution to pancreatic cancer, the crucial roles of autophagy and glycolysis in the progression of pancreatic cancer should be considered. In our previous study, miR-7 was identified to repress autophagy through the upregulation of LKB1-AMPK-mTOR signaling and directly targeting the stages of autophagy induction and vesicle elongation to reduce the supply of intracellular glucose to glycolysis metabolism [9]. Thereupon, in this study, we detected the LC3 and LDHA in the tissue microarray and observed that stronger activity of autophagy and glycolysis were exhibited in pancreatic cancers than normal tissues. As a result, miR-7 was significantly downregulated in pancreatic cancer compared with matched normal tissue. In addition, the expression levels of the majority of glycolysis metabolite-related genes were highly elevated in 178 primary human PDAC tumors in early stages compared to normal pancreatic tissues by TCGA database. This suggested that reprogrammed metabolic homeostasis and autophagy are crucial for pancreatic cancer proliferation and survival. We concluded that miR-7 might interfere with autophagy to impair glycolysis in pancreatic cancer patient.

Currently, gemcitabine is the standard chemotherapy used as the first-line treatment for patients with advanced pancreatic cancer. Nevertheless, the survival extension is only marginal [20]. The failure of effective chemotherapy results in high mortality in pancreatic cancer patients. Thus, understanding the mechanism of drug resistance and developing the biomarker for the prediction of the therapy efficacy would improve the outcome of the chemotherapy. Autophagy acts as a survival mechanism under conditions of stress, maintaining cellular integrity by regenerating metabolic precursors [21]. Recent studies found pancreatic cancer cells could escape gemcitabine-mediated cell apoptosis via activating autophagy. Autophagy blockade sensitized pancreatic cancer cells to gemcitabine [22–24]. Therefore, combination of gemcitabine and pharmacological autophagy inhibitor is a promising therapeutic strategy for pancreatic cancer. In addition, substantial evidences demonstrated the mechanistic link between aberrant expression of glycolytic enzymes and development of drug resistance in cancer cells through the upregulation of survival signaling. Further targeting the Warburg effect could overcome gemcitabine resistance in pancreatic cancer cells [25]. Note that miR-7 could repress the activity of Warburg effect by targeting autophagy to reduce the glucose supply in pancreatic cancer in our previous study [9]. In the present study, it was shown that miR-7 expression was dramatically decreased in the plasma of patients with gemcitabine-resistant pancreatic cancer and *in vitro* upregulation of miR-7 significantly enhanced the sensitivity of pancreatic cancer cells to gemcitabine. These data suggested that miR-7 might potentiate the tumoricidal effect of gemcitabine via targeting autophagy to impair glycolysis. Hence, miR-7 could be a predictive biomarker for the chemotherapy efficacy in pancreatic cancer.

## 5. Conclusions

Briefly, our study was the first time to systematically interrogate the function on autophagy and glycolysis and the clinical significance of miR-7 in pancreatic cancer. We showed that miR-7 was significantly downregulated in pancreatic cancer and that decreased miR-7 expression levels indicated poor prognosis of pancreatic cancer patients. Moreover, our results implicated miR-7 as a prognostic marker for gemcitabine-based chemotherapy. Mechanistically, our finding suggested clinical significance of miR-7 was involved in its interference effect on autophagy and glycolysis in pancreatic cancer. Thus, we concluded that miR-7 was a promising biomarker for pancreatic cancer, and targeting miR-7 might be a potential therapeutic strategy for the treatment of pancreatic cancer patients.

## Figures and Tables

**Table 1:** The relationship between miR-7 expression and clinicopathologic characteristics in pancreatic cancer patients.

Clinicopathologic variable	miR-7		*P* value
	Low expression	High expression	
Age (y)			0.637
≤50	7	11	
>50	30	52	
Gender			0.855
Female	12	25	
Male	25	38	
Tumor size (cm)^[a]^			0.645
≤2	0	2	
>2	37	59	
Tumor differentiation			0.015^∗^
Well/moderate	19	49	
Poor	18	14	
TNM stage^[b]^			0.028^∗^
I+II	35	62	
III+IV	2	0	
Lymph node metastasis^[c]^			0.930
Negative	20	31	
Positive	15	24	
Distant metastasis			0.011^∗^
Negative	35	63	
Positive	2	0	
Lymphovascular invasion			0.875
Absent	21	37	
Present	16	26	

^∗^Statistically significant (*P* < 0.05). ^[a]^2 patients' information missing; ^[b]^1 patient's information missing; ^[c]^10 patients' information missing.

**Table 2:** Multivariate analysis of factors associated with overall survival in pancreatic cancer patients.

Variable	HR (95% CI)	*P* value
Age	1.003 (0.981-1.025)	0.817
Gender	0.995 (0.599-1.653)	0.984
Tumor size	2.992 (0.710-12.608)	0.135
Tumor differentiation	0.803 (0.463-1.390)	0.433
TNM stage	6.018 (1.731-23.550)	0.010^∗^
Lymph node metastasis	0.996 (0.482-1.313)	0.372
Distant metastasis	11.659 (2.038-32.516)	0.001^∗^
miR-7	0.325 (0.198-0.531)	0.000^∗^

HR: hazard ratio; CI: confidence interval. ^∗^Statistically significant (*P* < 0.05).

## Data Availability

All data generated or analyzed during this study are included in this published article.

## Abbreviations

ciRS-7: Circular RNA sponge for miR-7
miRNA: MicroRNA
PDAC: Pancreatic ductal adenocarcinoma
TCGA: The Cancer Genome Atlas.
